# Surveillance of ARV safety in pregnancy and breastfeeding: towards a new framework

**DOI:** 10.1002/jia2.25922

**Published:** 2022-07-19

**Authors:** Françoise Renaud, Lynne M. Mofenson, Charlotte Bakker, Helen Dolk, Valeriane Leroy, Angelina Namiba, Leyla Sahin, Roger Shapiro, Amy Slogrove, Claire Thorne, Marissa Vicari, Daniel Low‐Beer, Meg Doherty

**Affiliations:** ^1^ Department of Global HIV Hepatitis and Sexually Transmitted Infections Programmes World Health Organization Geneva Switzerland; ^2^ Research Department Elizabeth Glaser Pediatric AIDS Foundation Washington DC USA; ^3^ Seconded National Expert Translational Sciences Office Scientific Evidence Generation Department European Medicines Agency Amsterdam The Netherlands; ^4^ EUROmediCAT Institute for Nursing and Health Research Ulster University Jordanstown United Kingdom; ^5^ Centre d'Epidémiologie et Recherche en Santé des POPulations (CERPOP) Inserm, Université de Toulouse Paul Sabatier Toulouse France; ^6^ 4M Network of Mentor Mothers London UK; ^7^ Division of Pediatrics and Maternal Health US Food and Drug Administration Silver Spring Maryland USA; ^8^ Harvard T.H. Chan School of Public Health Boston Massachusetts USA; ^9^ Department of Paediatrics and Child Health Faculty of Medicine and Health Sciences Stellenbosch University Worcester South Africa; ^10^ Great Ormond Street Institute of Child Health University College London London UK; ^11^ HIV Programmes and Advocacy Department International AIDS Society Geneva Switzerland

**Keywords:** HIV, antiretrovirals, pregnancy, safety, adverse pregnancy outcomes, surveillance

## Abstract

**Introduction:**

As new antiretrovirals (ARVs), including long‐acting ARVs for treatment and prevention, are approved and introduced, surveillance during pregnancy must become the safety net for evaluating birth outcomes, especially those that are rare and require large numbers of observations. Historically, drug pharmacovigilance in pregnancy has been limited and fragmented between different data sources, resulting in inadequate data to assess risk. The International Maternal Pediatric Adolescent AIDS Clinical Trials Network and World Health Organization convened a Workshop which reviewed strengths and weaknesses of existing programs and discussed an improved framework to integrate existing safety data sources and promote harmonization and digitalization.

**Discussion:**

This paper highlights that although robust sources of safety data and surveillance programs exist, key challenges remain, including unknown denominators, reporting bias, under‐reporting (e.g. in voluntary registries), few data sources from resource‐limited settings (most are in North America and Europe), incomplete or inaccurate data (e.g. within routine medical records). However, recent experiences (e.g. with safety signals) and current innovations (e.g. electronic record use in resource‐limited settings and defining adverse outcomes) provide momentum and building blocks for a new framework for active surveillance of ARV safety in pregnancy. A public health approach should be taken using data from existing sources, including registries of pregnancy ARV exposure and birth defects; observational surveillance and cohort studies; clinical trials; and real‐world databases. Key facilitators are harmonization and standardization of outcomes, sharing of materials and tools, and data linkages between programs. Other key facilitators include the development of guidance to estimate sample size and duration of surveillance, ensuring strategic geographic diversity, bringing partners together to share information and engaging the community of women living with HIV.

**Conclusions:**

Looking ahead, critical steps to safely introduce new ARVs include (1) adopting harmonized standards for measuring adverse maternal, birth and infant outcomes; (2) establishing surveillance centres of excellence in areas with high HIV prevalence with harmonized data collection and optimized electronic health records linking maternal/infant data; and (3) creating targets and evaluation goals for reporting progress on implementation and quality of surveillance in pregnancy. The platform will be leveraged to ensure that appropriate contributions and strategic actions by relevant stakeholders are implemented.

## 1 INTRODUCTION

Data on new antiretroviral (ARV) drugs in pregnancy are often delayed until years after initial approval [1, 2]. Additionally, the most vulnerable period for adverse foetal effects is in the early first trimester of drug exposure. With life‐long treatment recommended for all people living with HIV, ARV exposure during embryogenesis is increasingly common among women of reproductive age living with HIV, given that pregnancy is usually only recognized a month or more after fertilization. While there is urgency to facilitate access to newer ARVs to pregnant women living with HIV more rapidly, detection of less common adverse outcomes in pregnancy, such as birth defects, requires the evaluation of large numbers of early pregnancy exposures after an ARV is introduced into populations [3]. As new ARVs are approved and introduced, active surveillance must become the safety net to evaluate birth outcomes, especially those which are rare.

The unexpected identification of a neural tube defect (NTD) safety signal with preconception dolutegravir (DTG) exposure in the Botswana Tsepamo birth defect surveillance outcomes study brought into sharp focus the need for reliable data on drug safety in pregnancy and improved surveillance systems in resource‐limited settings to evaluate the safety of new drugs that will be widely used by women of reproductive potential [4, 5]. Safety data become even more critical as potential ARV exposure increases with pre‐exposure prophylaxis use among HIV‐negative women at risk of acquiring HIV and the availability of long‐acting ARV drugs, such as injectable cabotegravir, for treatment or prevention, with drug levels which persist for up to a year post‐injection [6].

The International Maternal Pediatric Adolescent AIDS Clinical Trials (IMPAACT) Network and World Health Organization (WHO) convened a Workshop which included a focus on surveillance. Building on the outcomes of the discussion, we propose an improved framework to integrate existing pregnancy safety data sources and promote harmonization and digitalization to improve drug safety surveillance.

## 2 DISCUSSION

### 2.1 Current state of pharmacovigilance in pregnancy—learning from the DTG NTD signal experience

In May 2018, WHO issued a statement regarding a potential safety signal on NTD risk in infants born to women living with HIV receiving DTG periconception, based on a preliminary analysis from the Botswana Tsepamo birth defect surveillance study [7]. During this study, the recommended first‐line antiretroviral therapy (ART) regimen in Botswana changed from being efavirenz (EFV) ‐ to DTG‐based, allowing the evaluation of birth defects identified through a body surface examination between ART regimens. A preliminary evaluation of birth defect data, done at the WHO's request for the 2018 Guidelines Development Group (GDG) meeting, found four NTDs in 426 periconception DTG exposures (0.94%) compared to 14 in 11,300 periconception non‐DTG exposures (0.12%) [4].

At that time, outside of the Tsepamo study, only limited and fragmented data were available related to birth defects with periconception ARV exposure. Exposure timing is critical in assessing the potential association with birth defects (e.g. the neural tube closes at 4 weeks post‐fertilization), but many studies do not distinguish between early (periconception) and late first trimester exposure. Having an accurate exposure denominator is critical to determine defect prevalence, but pharmaceutical company and regulatory agency pharmacovigilance databases are limited to spontaneous adverse outcome reports and collect limited data, making it difficult to discern duplicate reports [8]. Registries, such as the Antiretroviral Pregnancy Registry (APR), collect prospective data from women enrolled prior to delivery, but rely on clinician voluntary reporting and have limited data on newer drugs, reducing statistical power to detect associations with rare pregnancy outcomes; while the APR is an international registry, 73% of reports come from the United States [9]. Cohort studies may be retrospective in nature and have few exposed mother–infant pairs within individual cohorts.

Therefore, there was no single database other than the Tsepamo study that contained sufficient prospective exposures to periconception DTG. The WHO Advisory Committee on Safety of Medicinal Products (ACSoMP) created a DTG Safety Subcommittee to provide ongoing evaluation of NTD risk with periconception DTG and other integrase inhibitors (InSTI). Rapid collection of published and unpublished data from pharmaceutical companies, regulatory agencies, existing registries, various cohort studies, clinical trials, country programs and other partners was undertaken, with frequent Subcommittee updates. By May 2019, ongoing surveillance from Tsepamo showed a lower NTD risk estimate with periconception DTG exposure (0.30%) [10, 11, 12]. The data provided to ACSoMP provided reassurance to the WHO GDG that NTD risk with DTG exposure was likely to be low enough to recommend DTG as the preferred ARV for HIV treatment in the 2019 WHO guidelines update, including in women of reproductive potential, with the support of risk/benefit analyses and civil society consultation and advocacy [13, 14, 15].

This experience demonstrated that there are multiple critical factors for safety signal evaluation, including the number of exposures needed to assess an initial signal; data quality, interpretability and comparability and need for longitudinal data over time; unbiased data sources capturing an adequate comparison group and denominator; data sharing across multiple data systems; analysis of potential drug benefits as well as risks; appropriate messaging of risks and benefits in pregnant women and those of reproductive potential; and involvement of the affected community in such messaging [5]. The Workshop prompted evaluation of what was needed for improved data collection and standardization, and led to the collaborative development of a new conceptual framework for active ARV safety surveillance in pregnancy.

### 2.2 Getting ready for new ARVs: data collection and standardization

Guidance for optimizing and standardizing surveillance data collection and reporting has been limited. Pursuing a harmonized approach to collecting exposure data and measuring relevant outcomes through surveillance systems can be facilitated by standardized definitions and data collection tools for a core set of exposure and outcome variables.

#### 2.2.1 Outcomes of interest

Birth outcomes with the greatest public health impact include preterm and very preterm birth, being small‐for‐gestational age (SGA) and very SGA, stillbirths, neonatal deaths prior to hospital discharge and birth defects based on surface examination findings at birth. Data suggest that adverse birth outcomes may differ by ART regimen [16, 17, 18], and that phase 3 pre‐approval studies, even if including pregnant women, will be too small to capture differences for some outcomes, especially stillbirths and congenital anomalies [19]. Thus, active surveillance systems need to capture these outcomes in a uniform manner.

Obtaining denominator data for anomalies identified on surface examination is critical to understand potential signals of drug‐related toxicity, and surveillance efforts will fail if only abnormal examinations are recorded. Outcome prevalence has a large impact on the denominator required to understand a signal, with rare events, such as NTDs, requiring surveillance of large numbers of exposures to gain precision.

#### 2.2.2 Standardization

Standardization both within and across surveillance sites is paramount. Data quality checks should confirm that all births and all events are recorded in the same manner (e.g. by cross‐checking logbooks to confirm denominator data), weekends and holidays need to be covered and periodic trainings provided to hospital staff regarding the correct conduct of a surface examination. While communication and cooperation with hospital staff is critical, surveillance cannot depend on hospital staff or local resources; sufficient resources should be provided to the surveillance sites as part of the central surveillance network funding to ensure success.

Gestational age determination poses the greatest challenge for standardization and should be determined by second trimester ultrasound whenever possible [20, 21, 22]. If not done, estimates based on reported last menstrual period (ideally recorded at the first antenatal visit) or fundal height estimations may be needed. Comparisons of preterm birth across sites must account for differences in gestational age determination, ideally stratifying analyses by site. The surface examination can identify up to 73% of congenital anomalies in a low‐cost, standardized manner [23]. These outcomes include NTDs and other rare major anomalies that may not occur in smaller studies prior to drug approval.

There have been some examples of efforts to standardize data collection in surveillance programs. In Europe, the ConcePTION consortium, begun in 2019, included 20 experts in complementary fields from 10 countries to select, identify and define core evidence elements to assess safety in pregnancy, followed by a stakeholder consultation with external experts [24]. The report includes core outcome data elements and addresses measurement of maternal exposure and the aetiological window for each outcome, confounders, study designs, analytical considerations, statistical power and quality assessment [25]; the European Health Data and Evidence Network is supporting adoption of the ConcePTION Common Data Model across Europe to create a federated network of data systems [26].

The DECIPHER Project (Data Evaluation and CIPHER Preparation for an HIV‐Exposed Uninfected Child Cohort) Project of the CIPHER Program of the International AIDS Society has completed development of *in utero* HIV and ARV exposure definitions that can be used globally, and is working on harmonization of birth outcome and neonatal morbidity and mortality definitions. The DECIPHER definitions incorporate a “level of certainty” approach, initially proposed by the Brighton Collaboration [27, 28, 29].

Use of consistent exposure and outcome definitions across a multitude of contexts can lessen surveillance method heterogeneity and enhance the understanding of similarities and differences in outcome rates across the globe. As surveillance systems will need to be leveraged for evaluation of rare outcomes, data pooling across surveillance systems will be greatly strengthened in terms of feasibility as well as scientific integrity through harmonized collection of a core set of exposure and outcome variables.

#### 2.2.3 Equity in global representation

With 90% of pregnant women living with HIV in Africa, it is imperative that pregnancy ARV safety surveillance occurs on this continent as well as other settings with concentrated HIV epidemics. ARV surveillance in pregnancy is most effective in areas with high HIV prevalence and mature ART programs, and where a high proportion of women with HIV are receiving ART from the time of conception; these preconditions allow efficient data accrual, capture of exposures potentially related to birth defects and the ability to compare infants with those concurrently or historically exposed to a variety of ART regimens. Surveillance program design should include considerations related to geography, data collection feasibility, exposure prevalence and standardization. Geographic differences that may impact outcomes include genetic differences in populations, health, nutrition and supplementation practices in pregnancy. For example, NTD susceptibility is impacted by folic acid supplementation and food folate supplementation, and drug effects associated with this pathway may be unmasked only in regions that lack supplementation or sufficient folic acid in the diet [30]. Therefore, the inclusion of several geographic regions in coordinated surveillance efforts is needed. Surveillance is best suited to regions where women deliver in hospitals, to complete denominator data for all deliveries. Lack of representation of non‐hospital deliveries is an inherent limitation of such surveillance.

Recent years have seen the emergence of electronic health‐related data systems in a number of HIV high prevalence countries that can assist surveillance efforts. Perhaps the most mature is in the Western Cape Province of South Africa, where approximately 15 years ago the provincial government invested in unique patient identifiers used across all public‐sector health services. With the support of research and other partners, the Western Cape Provincial Health Data Centre integrates multiple electronic sources of data at an individual level to identify healthcare system encounters as well as disease episodes, such as pregnancy or HIV [31, 32]. Through the US NIH‐funded International Epidemiology Database to Evaluate AIDS (IeDEA) consortium and funding from the Gates Foundation, pilot sentinel birth outcomes surveillance has been set up in the Western Cape (Western Cape Pregnancy Exposure Registry) and at the Moi Teaching and Referral Hospital in western Kenya.

### 2.3 Engaging with communities on surveillance of ART effects on pregnancy

The affected community is a critical partner in surveillance using the principles of the Greater Involvement of People Living with HIV (GIPA). Several actions to strengthen community engagement in surveillance were identified [33]. Working with and strengthening existing community structures represents an opportunity to improve health literacy regarding surveillance and potentially develop innovative surveillance opportunities (e.g. for home deliveries, through community‐based monitoring). The community of women living with HIV can take a leading role in developing community literacy on surveillance, helping researchers translate jargon into plain, age‐ and culturally appropriate language that speaks to people of reproductive potential. For all the above actions, it is important to ensure that funding is available to enable meaningful engagement with the community of women living with HIV, support existing community structures and improve surveillance literacy.

### 2.4 A new collaborative framework for active surveillance of ARVS in pregnancy

Collaboration and data sharing are particularly critical for active surveillance of potential adverse outcomes associated with ARVs in pregnancy, as such occurrences may be rare and no one region/study is likely to have sufficient resources or numbers of pregnancy outcomes to answer the full scope of safety questions. Additionally, there are multiple challenges to establishing pharmacovigilance in low‐ and middle‐income settings [34].

**Figure 1:**
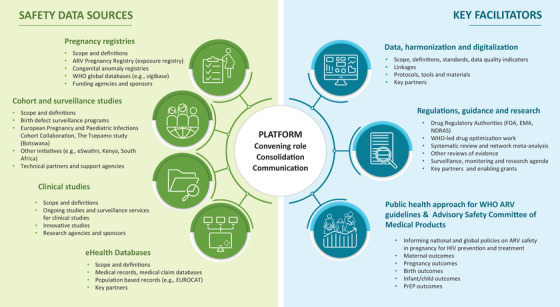
New collaborative conceptual framework for surveillance of safety of ARVs in pregnancy. Abbreviations: ARV, antiretrovirals; WHO, World Health Organization; EUROCAT, European Network For The Surveillance Of Congenital Anomalies; FDA, U.S. Food and Drugs Administration; EMA, European Medicines Agency; NDRAS, National Drug Regulatory Authorities.

Figure 1 shows a new collaborative conceptual framework for active surveillance of ARV safety in pregnancy developed during the Workshop. Key principles are: (1) improving the use of existing programs and fostering innovations; (2) building collaboration, harmonization and linkages between surveillance networks; (3) matching a timeline for surveillance system data analysis and data sharing to ensure they are informing ARV treatment and prevention policies within a public health response (i.e. WHO‐consolidated guidelines on HIV [35]); and (4) identification and collaborative evaluation of a signal when identified.

A convening organization (i.e. WHO) could adopt a modular, standardized platform for adverse pregnancy outcome surveillance, working with Ministries of Health and Centres of Excellence, to ensure standardization of data collection; promote surveillance innovations between partners; and bring partners together to share information, with engagement of the community of women living with HIV.

Surveillance data would come from existing partners, including pregnancy ARV exposure registries and birth defect registries; observational surveillance and cohort studies; clinical trials; and real‐world program data. Appendix S1 provides an inventory of existing sources of ARV safety in pregnancy.

Key facilitators are harmonization/standardization of outcomes between surveillance programs, sharing of materials and tools, and linkages between programs. Data quality indicators can also be important tools. For example, it should be possible to estimate the approximate expected number of pregnancy exposures each year (per country) and report the number of exposed pregnancies with known and unknown outcomes included in safety monitoring. A low number of exposed pregnancies with known outcomes compared to those expected suggests that improved monitoring is needed. EUROCAT has data quality indicators for birth defect registries, which identify the strengths and weaknesses of different registries so that focused data quality improvement can occur [36].

Other facilitators include the involvement of regulators (e.g. US Food and Drug Administration and European Medicines Agency) and other groups involved in prioritization of new drugs in pregnant populations. Data review would include systematic literature and abstract review along with available surveillance data, with meta‐analyses when possible. Guidance for sample size and surveillance duration is needed that will also ensure strategic geographic diversity to account for different birth defect background rates and rates of comorbidities, supplement use and other key factors.

Health system innovation in Africa has included the implementation of unique healthcare identifiers and development of electronic birth registers and patient‐tracking systems. The majority have been developed as national Ministries of Health initiatives with support from external academic, research or funding partners, but most remain restricted to pilot sites, sentinel surveillance sites or centres of excellence, with few upscaled to cover entire provinces, states or countries. Few have matured to include routine electronic data collection at the point of care, and rely on human resources for later data capture into an electronic system. There is a tension between financing immediate essential health supplies and financing long‐term data systems. There is a window of opportunity to advocate for and support integration of elements critical to pregnancy ARV safety surveillance, including individual‐level identification of pregnancy‐episodes and their outcomes, HIV status, medications received and mother–child linkage. Success of integrating pregnancy ARV safety surveillance into provincial or national routine electronic health system databases can be facilitated by consistent, reliable, long‐term external stakeholder financial and technical support to province or country‐owned electronic health systems. A public health approach should be adopted, with surveillance designed to inform WHO ARV guidelines and WHO ASCoMP on effects of ARV in pregnancy on maternal, pregnancy and neonatal outcomes, and develop effective messaging and dissemination to the community.

## 3 CONCLUSIONS

This paper advocates for the establishment of an improved collaborative framework for the surveillance of ARV safety in pregnancy. Looking ahead, the following steps will be critical to adopting a common framework for active surveillance: (1) implementing harmonized standards; (2) establishing surveillance centres of excellence in areas with high HIV prevalence, with harmonized data collection procedures and optimized electronic health records that link maternal/infant data; and (3) creating targets, monitoring and evaluation goals for reporting progress on implementation and quality of surveillance in pregnancy. Equitable access to better HIV medicines for pregnant women can only be achieved with stronger, broader and collaborative surveillance.

## COMPETING INTERESTS

LM has received funding from World Health Organization as consultant on antiretroviral drugs in pregnancy; Elisabeth Glazer Pediatric AIDS Foundation is receiving research funding from ViiV for a pilot birth surveillance program in Eswatini. MV reported that CIPHER is funded by grants from ViiV Healthcare, Janssen, Viatris and Merck. CT has received grant funding from ViiV Healthcare (via Penta Foundation). Other authors did not report competing interests.

## AUTHORS’ CONTRIBUTIONS

All authors participated in the workgroup and provided input for the paper.

## Supporting information


**Appendix S1**. Sources of ARV safety data in pregnancy and potential innovations
