# Supplementary material for: Surveillance of ARV safety in pregnancy and breastfeeding: towards a new framework
Source: J Int AIDS Soc. 2022 Jul 19;25(Suppl 2):e25922. doi: 10.1002/jia2.25922 (PMC9294858; doi:10.1002/jia2.25922)
Supplement: Supplementary file 1 — Appendix S1. Sources of ARV safety data in pregnancy and potential innovations [file JIA2-25-e25922-s001.docx]

**Electronic Appendix 1. Sources of ARV safety data in pregnancy and potential innovations**

***Antiretroviral Pregnancy Registry (www.apregistry.com):*** The Antiretroviral Pregnancy Registry (APR), was initiated in 1989; it currently includes 59 brand and 111 generic ARV formulations (1). It is a voluntary, prospective, exposure-registration, observational international study designed to collect and evaluate data on the outcomes of pregnancy exposures to ARV products and is intended to provide an early signal of any major teratogenic effects associated with a prenatal exposure to ARVs monitored through the registry. An independent expert Advisory Committee reviews data on birth defects reported to the APR on a 6-monthly basis and establishes a consensus regarding results.

***Observational Cohort Studies:*** Consented, prospective cohort studies have been major contributors on ARV safety in pregnancy. For example, the European Pregnancy and Paediatric Infections Cohort Collaboration (EPPICC) is an international network of 17 cohort studies that prospectively follow pregnant women with HIV and their children from 15 countries across Europe and Thailand; the cohorts contribute individual-level demographic, clinical, laboratory and treatment-related data, which are pseudo-anonymised and pooled electronically using a modified HIV Cohorts Data Exchange Protocol (HICDEP, [www.hicdep.org](http://www.hicdep.org)) (2,3).

***National HIV in Pregnancy Surveillance Cohorts****:* Some national HIV surveillance programs include a specific focus on HIV in pregnancy. In the United Kingdom, surveillance of all pregnant women with HIV and their infants is embedded within the National Health Service (NHS). A virtual cohort was created to “flag” children born to diagnosed mothers living with HIV, with secure event notifications sent to the surveillance service annually (4). In France, the French National Research Agency on HIV and Hepatitis (ANRS) pharmacovigilance system was established, to routinely notify all adverse events, including pregnancy outcomes occurring in the conduct of interventional and observational clinical research studies involving people living with HIV (5). In Canada, funded by the Public Health Agency of Canada, the Canadian Perinatal Surveillance Program (CPHSP) is conducted in 22 sites reporting on data from mother-infant dyads (6).

***Birth Surveillance Studies:*** The Tsepamo Birth Surveillance study in Botswana uses hospital-based delivery data on birth defects (7). A Research Assistant abstracts data from the obstetric record at the time of delivery from all women delivering live or stillbirths at study sites. Midwives conduct surface examination for birth defects as part of routine care for all infants. There are attempts to set up similar studies in Eswatini, Kenya and South Africa (8); pilot projects had been conducted in Uganda and Malawi (9).

***Population-Based Birth Defect Registries:*** Population-based birth defect registries are not specific to HIV and register all birth defects in defined geographical populations. Registries use multiple sources for diagnostic information validation, since it is important that birth defect ascertainment be complete and accurate. Case-control studies can be performed using the registry data, and some registries can be linked to maternal and birth records to conduct population cohort studies. In the United States, the birth defect registries participate in case-control surveillance selecting non-malformed controls for each defect case (NBDPS and BD-Steps studies, www.bdsteps.org).

***Pharmacovigilance Databases (Regulatory Authorities, Pharmaceutical Companies):*** There are several pharmacovigilance databases that collect adverse drug reactions (ADR) data for regulatory authorities, such as the European Medicines Authority (EMA) and US Food and Drug Administration (FDA) and pharmaceutical companies must collect safety data post-licensure on pregnancy outcomes with new ARV use in pregnancy as a requirement of regulatory agencies. There are several limitations when using these databases (10), notably the type and format of safety data varies by database, the duplication of reports, the lack of denominator and the risk of reporting bias, especially after a signal has been identified.

***Electronic Health (eHealth) Databases***

Recent years have seen the emergence of electronic health-related data systems in a number of HIV high prevalence countries. The most mature is the Western Cape Provincial Health Data Centre (WCPHDC) in South Africa which, using a unique patient identifiers, integrates multiple electronic sources of individual level data to identify healthcare system encounters and disease episodes (11). The WCPHDC has high ascertainment of pregnancies and their outcomes occurring in the public sector, HIV status with linked dispensed ART and linkage of mother-infant unique identifiers for longitudinal monitoring of HIV-exposed children (12).

There have been additional digital health system development in sub-Saharan Africa (SSA), with 41 of 54 African countries having national digital health strategies, and high-level recognition of the need to support eHealth innovations (13-15). A recent scoping review of maternal and neonatal data collection systems for maternal vaccine safety assessment in LMICs identified eight systems where active data collection on an individual mother-neonate level could be carried out, seven of which could link with external data sources and six with national health system databases, including the INDEPTH network of health and demographic surveillance systems (HDSS) in SSA and Asia and SmartCare in Zambia (16).

**References**

1. Antiretroviral Pregnancy Registry Steering Committee. Antiretroviral Pregnancy Registry Interim Report for 1 January 1989 through 31 July 2021. Wilmington, NC: Registry Coordinating Center; 2021. Available from URL: [www.APRegistry.com](http://www.APRegistry.com).
2. Favarato G, Bailey H, Burns F et al. Migrant women living with HIV in Europe: are they facting inequalities in the prevention of mother-to-child transmission of HIV?: the European Pregnancy and Paediatric HIV Cohort Collaboration (EPPICC) study group in EuroCoord. Eur J Public Health. 2018;28:55-60.
3. Kjaer J, Ledergerber B. HIV cohort collaborations: proposal for harmonization of data exchange. Antivir Ther. 2004;9:631-3.
4. Thorne C, Tookey P. Strategies for monitoring outcomes in HIV-exposed uninfected children in the United Kingdom. Front Immunol. 2016;7:185.
5. Saint-Lary L, Diallo A, de Monteynard L, et al. In utero exposure to antiretroviral drugs and pregnancy outcomes: analysis of the French ANRS pharmacovigilance database. British J Clin Pharmacol. 2021 (in press).
6. Forbes JC, Alimenti AM, Singer J et al. A national review of vertical HIV transmission. AIDS 2012;26:757-763.
7. Zash R, Holmes L, Diseko M, et al. Neural-tube defects and antiretroviral treatment regimens in Botswana. N Engl J Med. 2019;381:827-40.
8. Mehta UC, van Schalkwyk C, Naidoo P et al. Birth outcomes following antiretroviral exposure during pregnancy: initial results from a pregnancy exposure registry in South Africa. South Afr J HIV Med. 2019;20:971.
9. Mumpe-Mwanja D, Barlow-Mosha L, Willamson D et al. A hospital-based birth defects surveillance system in Kampala Uganda. BMC Pregnancy Childbirth. 2019;19:372.
10. Van De Ven NS, Pozniak AL, Levi JA et al. Analysis of pharmacovigilance databases for dolutegravir safety in pregnancy. Clin Infect Dis. 2020;70:2599-2606.
11. Boulle A, Heekes A, Tiffin N et al. Data Centre Profile: the provincial health data centre of the Western Cape Province, South Africa. Int J Popul Data Sci. 2019;4:1143.
12. Heekes A, Tiffin N, Dane P, Mutemaringa T, Smith M, Zinyakatira N, et al. Self-enrolment antenatal health promotion data as an adjunct to maternal clinical information systems in the Western Cape Province of South Africa. BMJ Glob Heal. 2018;3:1–9.
13. Holst C, Sukums F, Radovanovic D et al. Sub-Saharan Africa – the new breeding ground for global digital health. Lancet. 2020; 2:e160-162.
14. Nsaghurwe A, Dwivedi V, Ndesanjo W et al. One country’s journey to interoperability: Tanzania’s experience developing and implementing a national health information exchange. BMC Med Inform Decision Making. 2021;21:139.
15. World Health Organization. WHO Guideline: Recommendations on digital interventions for health system strengthening. 2019. URL: <https://apps.who.int/iris/bitstream/handle/10665/311941/9789241550505-eng.pdf?ua=1>
16. Berrueta M, Ciapponi A, Bardach A et al. Maternal and neonatal data collection systems in low- and middle-income countries for maternal vaccines active safety surveillance systems: a scoping review. BMC Pregnancy Childbirth. 2021;21:217.
